# Long-term outcomes of patients with IgA nephropathy in the German CKD cohort

**DOI:** 10.1093/ckj/sfae230

**Published:** 2024-07-22

**Authors:** Eleni Stamellou, Jennifer Nadal, Bruce Hendry, Alex Mercer, Claudia Seikrit, Wibke Bechtel-Walz, Matthias Schmid, Marcus J Moeller, Mario Schiffer, Kai-Uwe Eckardt, Rafael Kramann, Jürgen Floege, the GCKD study investigators, Kai-Uwe Eckardt, Heike Meiselbach, Markus P Schneider, Mario Schiffer, Hans-Ulrich Prokosch, Barbara Bärthlein, Andreas Beck, André Reis, Arif B Ekici, Susanne Becker, Ulrike Alberth-Schmidt, Sabine Marschall, Anke Weigel, Gerd Walz, Anna Köttgen, Ulla T Schultheiß, Fruzsina Kotsis, Simone Meder, Erna Mitsch, Ursula Reinhard, Jürgen Floege, Turgay Saritas, Elke Schaeffner, Seema Baid-Agrawal, Kerstin Theisen, Kai Schmidt-Ott, Martin Zeier, Claudia Sommerer, Mehtap Aykac, Gunter Wolf, Martin Busch, Andi Steiner, Thomas Sitter, Christoph Wanner, Vera Krane, Britta Bauer, Florian Kronenberg, Julia Raschenberger, Barbara Kollerits, Lukas Forer, Sebastian Schönherr, Hansi Weissensteiner, Peter Oefner, Wolfram Gronwald, Matthias Schmid, Jennifer Nadal

**Affiliations:** Division of Nephrology and Clinical Immunology, RWTH Aachen University Hospital, Aachen, Germany; Department of Nephrology, School of Medicine, University of Ioannina, Ioannina, Greece; Department of Medical Biometry, Informatics and Epidemiology, Faculty of Medicine, University Hospital Bonn, Bonn, Germany; Travere Therapeutics, San Diego, CA, USA; JAMCO Pharma Consulting, Stockholm, Sweden; Division of Nephrology and Clinical Immunology, RWTH Aachen University Hospital, Aachen, Germany; Department of Medicine IV, University Medical Center, Faculty of Medicine, University of Freiburg, Freiburg, Germany; Berta‐Ottenstein Program, Faculty of Medicine, University of Freiburg, Freiburg, Germany; Department of Medical Biometry, Informatics and Epidemiology, Faculty of Medicine, University Hospital Bonn, Bonn, Germany; Division of Nephrology and Clinical Immunology, RWTH Aachen University Hospital, Aachen, Germany; Department of Nephrology and Hypertension, Friedrich-Alexander-Universität Erlangen-Nürnberg, Erlangen, Germany; Department of Nephrology and Hypertension, Friedrich-Alexander-Universität Erlangen-Nürnberg, Erlangen, Germany; Department of Nephrology and Medical Intensive Care, Charité – Universitätsmedizin Berlin, Berlin, Germany; Division of Nephrology and Clinical Immunology, RWTH Aachen University Hospital, Aachen, Germany; Division of Nephrology and Clinical Immunology, RWTH Aachen University Hospital, Aachen, Germany; Department of Cardiology, RWTH Aachen University Hospital, Aachen, Germany

**Keywords:** albuminuria, CKD, glomerulonephritis, IgA nephropathy, proteinuria

## Abstract

**Background:**

The importance of albuminuria as opposed to proteinuria in predicting kidney outcomes in primary immunoglobulin A nephropathy (IgAN) is not well established.

**Methods:**

From 2010 to 2012, 421 patients with biopsy-proven IgAN have been enrolled into the German Chronic Kidney Disease (GCKD) cohort, a prospective observational cohort study (*N* = 5217). Adjudicated endpoints include a composite kidney endpoint (CKE) consisting of eGFR decline >40%, eGFR <15 ml/min/1.73 m^2^ and initiation of kidney replacement therapy; the individual components of the CKE; and combined major adverse cardiac events (MACE), including non-fatal myocardial infarction, non-fatal stroke and all-cause mortality. The associations between the incidence of CKE and baseline factors, including demographics, laboratory values and comorbidities were analysed using the Cox proportional hazards regression model.

**Results:**

The mean age of IgAN patients at baseline was 51.6 years (± 13.6) and 67% were male. The patient-reported duration of disease at baseline was 5.9 ± 8.1 years. Baseline median urine albumin:creatinine ratio (UACR) was 0.4 g/g [interquartile range (IQR) 0.1–0.8] and mean eGFR was 52.5 ± 22.4 ml/min/1.73 m^2^. Over a follow-up of 6.5 years, 64 (15.2%) patients experienced a >40% eGFR decline, 3 (0.7%) reached eGFR <15 ml/min/1.73 m^2^, 53 (12.6%) initiated kidney replacement therapy and 28% of the patients experienced the CKE. Albuminuria, with reference to <0.1 g/g, was most associated with CKE. Hazard ratios (HRs) at UACRs of 0.1–0.6 g/g, 0.6–1.4 g/g, 1.4–2.2 g/g and >2.2 g/g were 2.03 [95% confidence interval (CI) 1.02–4.05], 3.8 (95% CI 1.92–7.5), 5.64 (95% CI 2.58–12.33) and 5.02 (95% CI 2.29–11-03), respectively. Regarding MACE, the presence of diabetes [HR 2.53 (95% CI 1.11–5.78)] was the most strongly associated factor, whereas UACR and eGFR did not show significant associations.

**Conclusion:**

In the GCKD IgAN subcohort, more than every fourth patient experienced a CKE event within 6.5 years. Our findings support the use of albuminuria as a surrogate to assess the risk of poor kidney outcomes.

KEY LEARNING POINTS **What was known:**

- Immunoglobulin A nephropathy (IgAN) is the most common primary glomerulonephritis worldwide and poses a significant risk of kidney failure over a patient's lifetime.
- Low-grade proteinuria (<1 g/day) has been recognized as prognostically relevant, with no benign course of IgAN in the presence of proteinuria.
- The urine albumin:creatinine ratio (UACR) offers higher specificity and sensitivity to changes in glomerular permeability compared with traditional proteinuria measurements.

**This study adds:**

- In the German Chronic Kidney Disease cohort, more than a quarter of patients with IgAN experienced a composite kidney endpoint event.
- Albuminuria was the strongest predictor of poor kidney outcomes.
- A substantial risk of kidney failure is already seen in patients with a UACR of 0.1–0.6 g/g.
- Relapse after partial or complete remission was relatively uncommon, occurring in only 3.1% of the cohort.

**Potential impact:**

- These findings support the use of albuminuria as a surrogate marker for assessing the risk of poor kidney outcomes in IgAN patients.
- The study emphasizes the importance of targeting albuminuria reduction in the clinical management of IgAN to improve long-term kidney outcomes.

## INTRODUCTION

Immunoglobulin A nephropathy (IgAN), the most common primary glomerulonephritis globally, presents a significant risk of kidney failure over a patient's lifetime [1]. The disease exhibits a remarkably heterogeneous course, ranging from mild forms with potential spontaneous remission to rapidly progressive forms [2, 3]. Overall, life expectancy is modestly reduced for patients with IgAN but is highly dependent on whether the disease progresses to kidney failure [4].

Prognostic assessment of IgAN currently mostly relies on the extent of proteinuria, estimated glomerular filtration rate (eGFR) and blood pressure (BP) control [5–10]. Increasing proteinuria is associated with an increased risk of both kidney failure and cardiovascular risk [11, 12]. There is now increasing evidence that even low-grade proteinuria (<1 g/day) is prognostically relevant and that there is no ‘benign course’ of IgAN as long as there is proteinuria [8, 13]. Recently, regulatory authorities have acknowledged the reduction in proteinuria as a surrogate endpoint for IgAN [14]. Traditionally, 24-h urine protein excretion (UPE) has been the standard for assessing proteinuria in randomized controlled trials (RCTs), but it is not without limitations [15]. Compared with proteinuria [either UPE or urine protein:creatinine ratio (UPCR)], the urine albumin:creatinine ratio (UACR) offers higher specificity and increased sensitivity to changes in glomerular permeability [16–19]. In two Chinese cohorts of patients with IgAN, UACR was found to better predict kidney failure compared with UPE and UPCR [20, 21].

In the present study, we analysed clinical data from patients with IgAN enrolled into the German Chronic Kidney Disease (GCKD) cohort, a national non-interventional cohort study with highly granular data and adjudicated endpoints. We sought to investigate their characteristics and outcomes and the association between albuminuria and the risk of cardiovascular and kidney outcomes.

## MATERIALS AND METHODS

### Study design and population

Between 2010 and 2012, the GCKD study enrolled 5217 participants of European ancestry ages 18–74 years under nephrological care with an eGFR of 30–60 ml/min/1.73 m^2^ (corresponding to CKD stages G3, A1–3) or an eGFR ≥60 ml/min/1.73 m^2^ in the presence of increased albuminuria in a spot urine sample (i.e. UACR >300 mg/g creatinine; corresponding to CKD stages G1–2, A3). The main exclusion criteria were non-European ancestry, active malignancy in the previous 2 years, previous transplantation, or heart failure (New York Heart Association class IV). All 421 participants with biopsy-proven IgAN as the leading cause of CKD were included in the analysis. It should be noted that the general inclusion criteria and methodology of the GCKD study were applicable to this specific IgAN cohort. The baseline date was defined as the first study visit. The median follow-up time after baseline was 6.5 years.

Every participant in the study gave written informed consent. The ethics committees of all nine German regional centres participating in the study approved the study. The study was carried out in accordance with approved guidelines and the Declaration of Helsinki. The study was registered in the German Clinical Trials Register (www.drks.de; DRKS00003971). The reporting guidelines of the Strengthening the Reporting of Observational Studies in Epidemiology were followed [22].

Details of the study enrolment and follow-up procedures have been described previously [23]. At baseline and follow-up study visits, trained and certified personnel used standardized questionnaires to obtain information about each patient's medical history, sociodemographic and lifestyle factors and medication intake. Further information about medical history and additional medical records were obtained from the treating nephrologists. All clinical measurements were performed according to predefined standard operating procedures.

Hypertension was defined as either systolic BP ≥140 mmHg or diastolic BP ≥90 mmHg or use of antihypertensive medication. Diabetes mellitus was defined as haemoglobin A1c ≥6.5% or the use of antidiabetic medication. Complete and partial remission were defined as UACR <0.1 g/g and UACR ≥0.1–<0.6 g/g, respectively. Relapse was defined as a single albuminuria value ≥0.6 g/g after any remission (complete or partial). Ever-smoker was defined as currently smoking or having smoked in the past. At baseline and follow-up visits, biomaterials including serum, plasma and urine were collected and transported frozen to a central biobank for future analyses following standard operating procedures. Blood and urine specimens were analysed in a central certified laboratory. Serum creatinine was analysed using an isotope dilution mass spectrometry traceable methodology (Creatinine Plus, Roche Diagnostics, Rotkreuz, Switzerland). Measures of kidney function included eGFR calculated with the Chronic Kidney Disease Epidemiology Collaboration (CKD-EPI) formula and UACR.

### Outcomes

Endpoints were continuously recorded based on hospital discharge letters and death certificates and were centrally adjudicated by experienced physicians. For this project, we included endpoints occurring over the first 6.5 years of follow-up. Adjudicated endpoints included a composite kidney endpoint (CKE) consisting of eGFR decline >40%, eGFR <15 ml/min/1.73 m^2^ and initiation of kidney replacement therapy, the individual components of the CKE and combined major adverse cardiac events (MACE), including non-fatal myocardial infarction, non-fatal stroke and all-cause mortality. All events until February 2022 (data export) were taken into account for the analysis.

### Statistical analysis

Continuous variables were expressed as means and standard deviations (SDs) in case of normally distributed variables or medians with interquartile ranges (IQRs) for non-normally distributed variables. Categorical variables were expressed as numbers with percentages. The eGFR slopes were evaluated in linear mixed effects models. Repeated measurements of a participant were included by random intercept and random slope term (i.e. random effects). The random intercept allows a participant-specific eGFR at baseline and the random slope reflects a participant-specific change in eGFR over time. All data were collected and managed using Askimed as a cloud-based web platform (https://www.askimed.com). Data extraction from Askimed was performed in February 2022. To identify determinants of eGFR levels, we used linear regression modelling, including the following baseline covariates: age, gender, body mass index (BMI), diabetes, systolic BP and UACR in categories as time dependent variables and presented the estimate (β) with 95% CIs.

Time-to-event outcomes were defined as follows: If patients failed to complete the 6-year follow-up period, censoring was performed at the time of the last follow-up, e.g. when participants were lost to follow-up or refused to further participate in the study. The Cox regression analyses for the specific outcomes (CKE and MACE) were conducted with a competing risks approach using cause-specific Cox regression. Confounding risk factors at baseline included age, sex, BMI, diabetes, systolic BP, eGFR (using the CKD-EPI 2009 equation) and as time-dependent UACR in categories. Estimates are presented as HRs with 95% CIs. Analyses were performed using SAS version 9.4 (SAS Institute, Cary, NC, USA).

## RESULTS

### Characteristics of the study population

The mean age at baseline was 51.6 ± 13.6 years and 67% (*n* = 282) were men (Table 1). The mean patient reported duration of the disease before enrolment was 5.9 ± 8.1 years. At baseline, the median UACR was 0.4 ± 0.1 g/g and the mean eGFR was 52.5 ± 22.4 ml/min/1.73 m^2^. Approximately 28% of the patients were in CKD stage 1 or 2 at baseline, 61.9% in CKD stage 3 and 10% in CKD stage 4 or 5. Regarding albuminuria, only 26.1% of patients had a UACR <0.1 g/g, while 60% had a UACR of 0.1–1.4 g/g and ≈13% had a UACR >1.4 g/g. Among the patients, 13.5% had diabetes at baseline and nearly all had hypertension (98%). At baseline, 92% of the patients were on therapy with an angiotensin-converting enzyme inhibitor or angiotensin receptor blocker and 15.6% were on dual treatment. A total of 15% received corticosteroids, while 6% were receiving immunosuppressive treatment other than steroids, with azathioprine being the most common. Systolic BP was 134 ± 17.3 mmHg and diastolic BP was 81.3 ± 11.2 mmHg.

**Table 1:** Demographics and clinical parameters at baseline.

		UACR categories (baseline)			
Variable	Total (*N* = 421)	≥0–<0.1 g/g [*n* = 110 (26.1%)]	≥0.1–<0.6 g/g [*n* = 158 (37.5%)]	≥0.6–<1.4 g/g [*n* = 99 (23.5%)]	≥1.4 g/g [*n* = 54 (12.9%)]
Sex, *n* (%)					
Male	282 (67)	67 (61)	118 (75)	61 (62)	36 67)
Female	139 (33)	43 (39)	40 (25)	38 (38)	18 (33)
Age (years), mean ± SD	51.6 ± 13.6	57.0 ± 11.9	51.8 ± 13.2	48.2 ± 14.4	45.9 ± 12.9
Duration of onset (years), mean ± SD	5.9 ± 8.1	8.8 ± 10.7	5.4 ± 7.0	4.7 ± 6.3	7.7 ± 2.3
BMI (kg/m^2^), mean ± SD	28 ± 5.2	28.9 ± 5.8	27.4 ± 4.5	27.9 ± 5.3	27.9 ± 5.6
Systolic BP (mmHg), mean ± SD	134.4 ± 17.3	131.2 ± 17.3	133.4 ± 16.4	135.2 ± 17.3	142.3 ± 17.6
Diastolic BP (mmHg), mean ± SD	81.3 ± 11.2	77.8 ± 10.9	80.6 ± 11.1	83.2 ± 10.7	87.2 ± 10.0
Smoking, *n* (%)					
Non-smoker	177 (42.2)	46 (42.2)	73 (46.2)	41 (41.4)	17 (32.1)
Former smoker	156 (37.2)	46 (42.2)	54 (34.2)	36 (36.4)	20 (37.7)
Current smoker	86 (20.5)	17 (15.6)	31 (19.6)	22 (22.2)	16 (30.2)
eGFR (CKD-EPI) (ml/min/1.73 m^2^), mean ± SD	52.5 ± 22.4	48.1 ± 14.8	53.5 ± 22.9	56.8 ± 28.2	50.4 ± 20.1
eGFR category, *n* (%)					
G1: CKD-EPI ≥90	38 (9)	1 (0.9)	17 (10.8)	17 (17.2)	3 (5.6)
G2: CKD-EPI ≥60–<90	80 (19)	26 (23.6)	28 (17.8)	14 (14.1)	12 (22.2)
G3a: CKD-EPI ≥45–<60	103 (24.5)	26 (23.6)	40 (25.5)	22 (22.2)	15 (27.8)
G3b: CKD-EPI ≥30–<45	157 (37.4)	49 (44.5)	55(35.0)	36 (36.4)	17 (31.5)
G4: CKD-EPI ≥15–<30	41 (9.8)	8 (7.3)	17 (10.8)	9 (9.1)	7 (13.0)
G5: CKD-EPI <15	1 (0.2)	0	0	1 (1.0)	0
Diabetes mellitus, *n* (%)	57 (13.5)	18 (31.6)	23 (40.3)	9 (15.8)	7 (12.4)
Hypertension, *n* (%)	413 (98.1)	106 (25.7)	157 (38.0)	97 (23.5)	53 (12.8)
CHD, *n* (%)	29 (6.9)	9 (31.0)	12 (41.4)	4 (13.8)	4 (13.8)
Antihypertensive therapy, *n* (%)	402 (96.4)	104 (25.9)	155 (38.6)	91 (22.5)	52 (12.9)
ACEi, *n* (%)	246 (59)	63 (25.6)	91 (37.0)	56 (22.8)	36 (14.6)
ARB, *n* (%)	205 (49.2)	47 (22.9)	88 (42.9)	47 (22.9)	23 (11.2)
Dihydropyridines (nifedipine-type), *n* (%)	149 (35.7)	44 (29.5)	50 (33.6)	29 (19.5)	26 (17.4)
Diuretics, *n* (%)	203 (48.7)	57 (28.1)	82 (40.4)	44 (21.7)	20 (9.6)
Thiazides, *n* (%)	103 (24.7)	24 (23.3)	45 (43.7)	26 (25.2)	8 (7.8)
Loop diuretics, *n* (%)	96 (23)	33 (34.4)	33 (34.4)	19 (19.8)	11 (11.4)
Aldosterone antagonists, *n* (%)	14 (3.4)	5 (35.7)	6 (42.9)	1 (7.1)	2 (14.3)
Statins, *n* (%)	186 (44.6)	43 (23.1)	74 (39.8)	39 (21.0)	30 (16.1)
Immunosuppressives, *n* (%)	25 (6)	7 (28.0)	9 (36.1)	5 (20.0)	4 (16.0)
Glucocorticoids, *n* (%)	63 (15.1)	18 (28.6)	14 (22.2)	18 (28.6)	13 (20.6)
Proton pump inhibitors, *n* (%)	87 (20.9)	30 (34.5)	24 (27.6)	21 (24.1)	12 (13.8)

ACEi: angiotensin-converting enzyme inhibitor; ARB: angiotensin II receptor blocker; CHD: coronary heart disease.

### Kidney outcomes

Over a follow-up of 6.5 years, 64 (15.2%) patients experienced a >40% eGFR decline, 3 (0.7%) reached an eGFR <15 ml/min, 53 (12.6%) initiated kidney replacement therapy and 28% experienced a CKE (Supplementary Table S1). Relapse after partial or complete remission was relatively uncommon, occurring in only 3.1% of the cohort.

**Figure 1:**
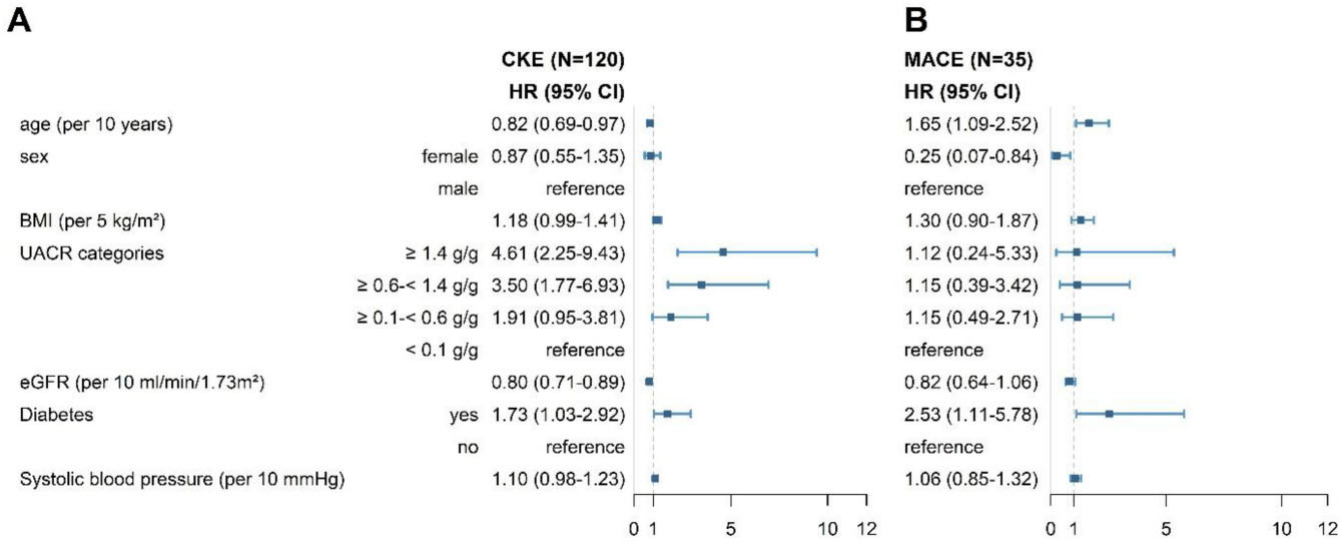
Forest plots of **(A)** CKE and **(B)** MACE showing HRs and 95% CIs.

Albuminuria, with reference to <0.1 g/g was most strongly associated with CKE. HRs at a UACR ≥0.1–0.6 g/g, ≥0.6–1.4 g/g, ≥1.4–2.2 g/g and ≥2.2 g/g were 2.03 (95% CI 1.02–4.05), 3.8 (95% CI 1.92–7.5), 5.64 (95% CI 2.58–12.33) and 5.02 (95% CI 2.29–11-03), respectively (Fig. 1A and Table 2). Each 10 ml/min higher eGFR at baseline was found to be protective against CKE [HR 0.80 (95% CI 0.71–0.89)]. A history of diabetes was found to be associated with an increased HR of 1.73 (95% CI 1.03–2.92), while each 10 years older age was associated with a decreased risk of CKE [HR 0.82 (95% CI 0.69–0.97)].

**Table 2:** Multivariate Cox model for predictors of CKE and MACE.

Variables	CKE (*n* = 120/421), HR (95% CI)	MACE (*n* = 35/421), HR (95% CI)
Age per 10-year increase (baseline)	0.82 (0.69–0.97)	1.65 (1.09–2.52)
Sex		
Female	0.87 (0.55–1.35)	0.25 (0.07–0.84)
Male	Reference	Reference
BMI per 5 kg/m^2^ (baseline) increase	1.18 (0.99–1.41)	1.30 (0.90–1.87)
UACR categories, g/g		
≥1.4	4.61 (2.25–9.43)	1.12 (0.24–5.33)
>0.6–<1.4	3.50 (1.77–6.93)	1.15 (0.39–3.42)
≥0.1–<0.6	1.91 (0.95–3.81)	1.15 (0.49–2.71)
≥0–<0.1	Reference	Reference
eGFR per 10 ml/min/1.73 m^2^ (baseline) increase	0.80 (0.71–0.89)	0.82 (0.64–1.06)
Diabetes mellitus		
Yes	1.73 (1.03–2.92)	2.53 (1.11–5.78)
No	Reference	Reference
Systolic BP (per 10 mmHg) increase	1.10 (0.98–1.23)	1.06 (0.85–1.32)

### Relationship between albuminuria, eGFR decline and kidney failure

**Figure 2:**
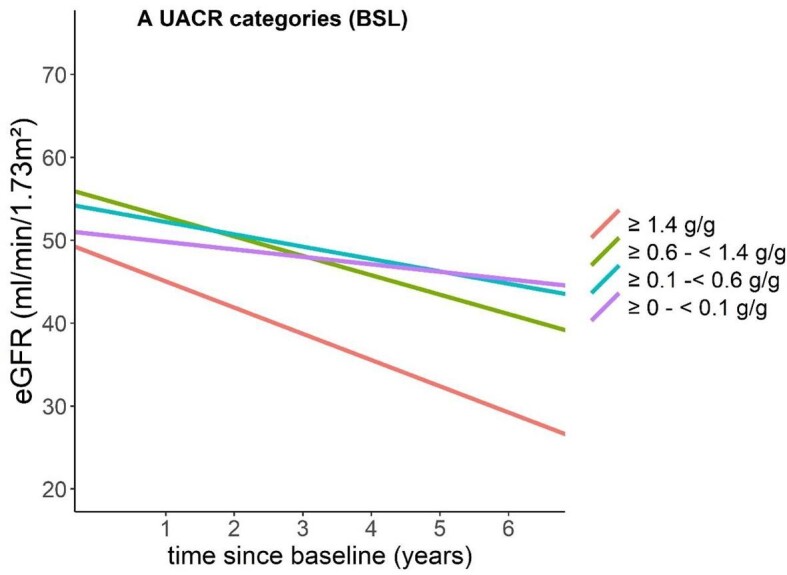
eGFR slopes according to UACR levels. Patients with higher baseline UACR levels (≥1.4 g/g) show a significantly steeper decline in eGFR over the 6-year follow-up period, indicating a greater risk of kidney function deterioration compared with those with lower UACR levels.

The rate of eGFR decline was greater among patients with albuminuria >1.4 g/g, with an average decrease of 3.16 ml/min/1.73 m^2^/year (Fig. 2 and Supplementary Table S2). Conversely, the lowest rate of decline was observed in patients with a UACR <0.1 g/g, at 0.90 ml/min/1.73 m^2^/year, indicating rather stable kidney function over time. Intermediate rates of eGFR decline were noted in the UACR categories of 0.6–<1.4 g/g and 0.1–<0.6 g/g, with slopes of −2.34 and −1.49 ml/min/1.73 m^2^/year, respectively.

**Figure 3:**
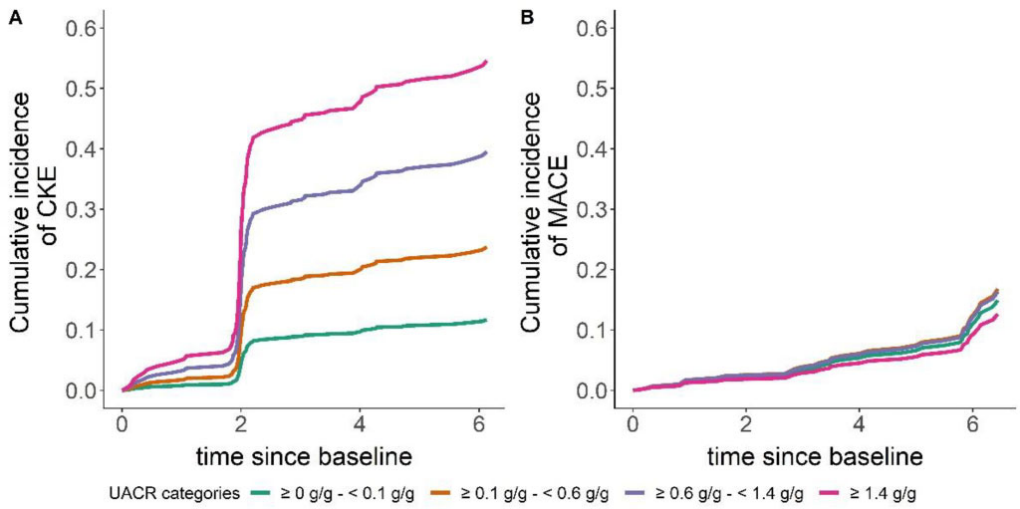
Cumulative incidence of **(A)** CKE and **(B)** MACE according to baseline UACR levels. The sudden increases in CKE incidence at 2 years (and subsequent smaller steps) are likely due to the fact that changes in eGFR were assessed for the first time at 2 years and then subsequently at 4 and 6 years.

Albuminuria from baseline over the total follow-up was analysed as a categorical variable, with higher albuminuria associated with worse kidney survival. Nearly 40% of individuals with a UACR of 0.6–1.4 g/g experienced CKE over the specified period, while notably 15% of individuals with a UACR of 0.1–0.6 experienced CKE (Fig. 3, Table 3).

**Table 3:** Cumulative incidence of CKE and MACE according to UACR categories.

	CKE, %				MACE, %			
UACR categories BSL	≥0–<0.1 g/g	≥0.1–<0.6 g/g	≥0.6–<1.4 g/g	≥1.4 g/g	≥0–<0.1 g/g	≥0.1–<0.6 g/g	≥0.6–<1.4 g/g	≥1.4 g/g
FU2	4.77	10.10	17.93	26.64	2.12	2.42	2.35	1.78
FU4	9.65	19.83	33.66	47.44	5.04	5.72	5.57	4.23
FU6	11.25	22.89	38.29	53.07	5.04	5.72	5.57	4.23

BSL: baseline; FU2: follow-up at 2 years; FU4: follow-up at 4 years; FU6: follow-up at 6 years.

The percentages indicate the proportion of patients within each UACR category who experienced CKE or MACE during the specified follow-up periods.

### Cardiovascular outcomes

During the follow-up period, 35 individuals experienced a first MACE (*n* = 19 patients with non-fatal myocardial infraction or stroke, *n* = 16 with all-cause mortality) (Supplementary  Table S3). A statistically significant association between age and MACE was found, where a 10-year increase in age was associated with a 1.65-fold higher hazard (95% CI 1.09–2.52) (Table 2, Fig. 1B). Additionally, the presence of diabetes was significantly associated with a 2.53-fold higher hazard of MACE (95% CI 1.11–5.78). Female gender was associated with a significantly lower hazard of MACE [HR 0.25 (95% CI 0.07–0.84)]. Other variables such as BMI, UACR, BP and eGFR did not show statistically significant associations with MACE in the analysis. Notably, ≈5% of individuals with a UACR of 0.6–1.4 g/g experienced MACE during the study period (Fig. 3, Table 3).

## DISCUSSION

The present study explored the clinical characteristics and outcomes of patients diagnosed with IgAN within the GCKD cohort. Over a 6.5-year follow-up period, 28% of patients with IgAN experienced a CKE. Relapse after partial or complete remission was relatively uncommon, occurring in only 3.1% of the cohort, suggesting that once remission is achieved, the risk of a significant increase in albuminuria remains low. Albuminuria was the strongest predictor of kidney failure, with higher levels correlated with increased risk.

Regarding relative risks, our findings indicate that compared with a UACR <0.1 g/g, any higher UACR level was independently associated with a greater rate of CKE. Specifically, we observed that patients with a UACR of 0.1–0.6 g/g had a 2.03-fold increased risk of experiencing a CKE. For those with a UACR of 0.6–1.4 g/g, the risk was 3.8 times higher, highlighting a significant linear association between elevated UACR levels and worse kidney outcomes. These findings are aligned with findings in 2 Chinese cohorts and a recently published Swedish cohort [8, 13, 24]. The recent Swedish study found a strong and incremental association between UACR and the risk of adverse kidney events. Conversely, as expected, higher baseline eGFR was protective. An eGFR decline was observed across all albuminuria categories, yet patients with <0.1 g/g of albuminuria demonstrated a more stable disease course, with a ‘physiological’ decline of <0.9 ml/min/1.73 m^2^/year.

Current phase 2 and 3 RCTs for IgAN use proteinuria as a surrogate endpoint, usually enrolling patients with baseline proteinuria >1 g/day (approximately equivalent to a UPCR of 0.88 g/g or a UACR of 0.6 g/g). Recently it was shown that ≈20% of patients with a time-averaged UPCR <0.44 g/g and 30% with a time-averaged UPCR of 0.44–<0.88 g/g progressed to kidney failure within 10 years of diagnosis [8]. Correspondingly, in our study, 15% of individuals with a UACR in the range of 0.1–0.6 g/g experienced CKE within <10 years of follow-up.

Regarding cardiovascular events and mortality, our analysis did not find any association between elevated UACR or lower eGFR. Research on the connection between proteinuria and cardiovascular events in IgAN is limited. A Canadian study reported a 10-year risk of 7.4%, with both proteinuria and lower eGFR linked to a higher incidence of cardiovascular events, though not specific to IgAN [25]. A recent US cohort study also found elevated proteinuria associated with higher cardiovascular disease risk and mortality [26]. One explanation could be our study's small sample size and the relatively short follow-up period of 6.5 years.

Currently, numerous new RCTs are investigating how to halt the progression of IgAN. This surge of RCTs is largely based on the recognition that a reduction of proteinuria in so-called high-risk patients is associated with improved outcomes and a slower rate of kidney function loss [14]. New drugs such as targeted release budesonide, sparsentan and potentially inhibitors targeting the APRIL (a proliferation-inducing ligand)/BAFF (B cell activating factor from the TNF family) signalling pathway, as well as complement activation, are undergoing evaluation, particularly in patients with high proteinuria [3, 27, 28]. However, there is a notable absence of IgAN patients with lower proteinuria levels in current RCTs. Based on our data, which suggest that renal risk continuously decreases with decreasing UACR, it will be important to study whether drugs that reduce high levels of proteinuria are also effective at reducing lower levels and if these effects translate to better kidney outcomes.

A limitation of our study is that patients were recruited into the study during their natural disease course and that data between recruitment into the cohort and disease diagnosis were not available. Second, despite the large size of the GCKD cohort, our sample size was relatively small, which may influence the statistical power of our results. Our analysis also did not include other clinical or histological parameters known to influence the progression of IgAN, such as the MEST-C score or the presence/absence of haematuria [29–31]. Additionally, given the fact that the pathophysiology of IgAN seems to be different between Asian and Caucasian populations, the fact that our study was limited to people with European ancestry may affect the generalizability of our findings. The participants were enrolled in Germany on the basis of prevalent non-dialysis-dependent CKD, thus there is a selection bias of participants owing to this defined study cohort. Further larger-scale studies with longer follow-up periods are warranted to validate our findings and further elucidate the factors influencing the prognosis of patients with IgAN. Third, we did not measure urine protein concentrations at baseline, which precludes a comparison with UACR.

The strengths of our analysis include that this cohort consists exclusively of patients treated by nephrologists, ensuring relatively standardized care. This minimizes potential cofounding variables arising from variations in medical management and highlights the clinical relevance of our results. Additionally, the GCKD cohort provides uniquely granular data related to patient demographics, medical history, treatment regimens and outcomes. Systematic endpoint adjudication further enhances outcome data accuracy and reduces bias. The use of standardized questionnaires to evaluate participants’ characteristics, in-person study visits conducted by trained study nurses, along with continuous evaluation of outcomes by experienced physicians following predefined criteria further enhances the reliability of our findings.

In summary, our study further supports the significant disease burden of IgAN. We confirm in a purely Caucasian population the critical importance of albuminuria as a modifiable risk factor in the management of IgAN. The need for targeted intervention in patients with elevated albuminuria is clear, as is the potential benefit of aggressive treatment strategies to maintain or improve renal function.

## Supplementary Material

sfae230_Supplemental_File

## Data Availability

The data underlying this article are available in the article and in its online supplementary material.
