# Supplementary material for: Long-term outcomes of patients with IgA nephropathy in the German CKD cohort
Source: Clin Kidney J. 2024 Jul 22;17(8):sfae230. doi: 10.1093/ckj/sfae230 (PMC11324945; doi:10.1093/ckj/sfae230)
Supplement: sfae230_Supplemental_File [file sfae230_supplemental_file.docx]

**Suppl. Tables**

| Suppl. Table 1. Incidence of CKE and MACE | | |
| --- | --- | --- |
| CKE | eGFR 40% decline | 64 (15.2%) |
|  | eGFR confirmed < 15 | 3 (0.7%) |
|  | ESKD | 53 (12.6%) |
| MACE | Nonfatal myocardial infraction and nonfatal stroke | 19(4.5%) |
|  | All-cause mortality | 16 (3.8%) |

| Suppl. Table 2. eGFR slope | | | |
| --- | --- | --- | --- |
|  |  | Intercept | Slope |
| UACR categories | ≥ 1.4 g/ | 48.20 | -3.16 |
|  | ≥ 0.6 g/g to < 1.4 g/g | 55.14 | -2.34 |
|  | ≥ 0.1 g/g to < 0.6 g/g | 53.71 | -1.49 |
|  | ≥ 0 g/g to < 0.1 g/g | 50.70 | -0.90 |
